# Melanin-templated rapid synthesis of silver nanostructures

**DOI:** 10.1186/1477-3155-12-18

**Published:** 2014-05-01

**Authors:** George Seghal Kiran, Asha Dhasayan, Anuj Nishanth Lipton, Joseph Selvin, Mariadhas Valan Arasu, Naif Abdullah Al-Dhabi

**Affiliations:** 1 Department of Food Science and Technology, Pondicherry University, Puducherry 605014, India; 2 Department of Microbiology, Bharathidasan University, Tiruchirappalli 620 024, India; 3 Department of Microbiology, Pondicherry University, Puducherry 605014, India; 4 Department of Botany and Microbiology, Addiriyah Chair for Environmental Studies, College of Science, King Saud University, P. O. Box 2455, Riyadh 11451, Saudi Arabia

**Keywords:** Marine *Nocardiopsis*, Melanin, Optimization, Silver nanostructures, Antimicrobial activity

## Abstract

**Background:**

As a potent antimicrobial agent, silver nanostructures have been used in nanosensors and nanomaterial-based assays for the detection of food relevant analytes such as organic molecules, aroma, chemical contaminants, gases and food borne pathogens. In addition silver based nanocomposites act as an antimicrobial for food packaging materials. In this prospective, the food grade melanin pigment extracted from sponge associated actinobacterium *Nocardiopsis alba* MSA10 and melanin mediated synthesis of silver nanostructures were studied. Based on the present findings, antimicrobial nanostructures can be developed against food pathogens for food industrial applications.

**Results:**

Briefly, the sponge associated actinobacterium *N. alba* MSA10 was screened and fermentation conditions were optimized for the production of melanin pigment. The Plackett-Burman design followed by a Box-Behnken design was developed to optimize the concentration of most significant factors for improved melanin yield. The antioxidant potential, reductive capabilities and physiochemical properties of *Nocardiopsis* melanin was characterized. The optimum production of melanin was attained with pH 7.5, temperature 35°C, salinity 2.5%, sucrose 25 g/L and tyrosine 12.5 g/L under submerged fermentation conditions. A highest melanin production of 3.4 mg/ml was reached with the optimization using Box-Behnken design. The purified melanin showed rapid reduction and stabilization of silver nanostructures. The melanin mediated process produced uniform and stable silver nanostructures with broad spectrum antimicrobial activity against food pathogens.

**Conclusions:**

The melanin pigment produced by *N. alba* MSA10 can be used for environmentally benign synthesis of silver nanostructures and can be useful for food packaging materials. The characteristics of broad spectrum of activity against food pathogens of silver nanostructures gives an insight for their potential applicability in incorporation of food packaging materials and antimicrobials for stored fruits and foods.

## Background

Silver particles/nanostructures have been used as an effective antimicrobial agent in food and beverage storage for a long time. Silver containing plastics had been incorporated in refrigerator liners and food storage containers [1-3]. FDA has been approved the use of silver based particles for disinfection purpose for the food contacting materials [4].

Silver based nanomaterials and nanocomposite can be devised for the easiest detection of commonly found food adulterants, chemical contaminants, allergens and any changes respond to environmental conditions etc. Silver nanoparticles incorporated cellulose pads are used to control the food pathogens from packed beef meat and reduce the microbial count in fresh cut melon [5]. Apart from this, silver nanoparticles slower the ripening times of stored fruits by catalyzing the destruction of ethylene gas and increase the shelf lives of stored fruits [5]. Several studies have demonstrated the efficacy of silver nanoparticles loaded packaging materials in campaigning against microbial growth in foods [5-8]. Nanostructured antimicrobials have a higher surface area-to-volume ratio than their microscale counterpart and their incorporation in food packaging systems are supposed to be particularly efficient in their activities against microbial cells [9]. The development of stable, mono dispersible, metallic silver nanostructures synthesis via reliable green synthesis has been an important aspect of current nanotechnology research. The aggregation of silver nanostructures and the insufficient stability of their dispersions lead to loss of their special nanoscale properties. Researchers employ polymer–assisted fabrication routes and various chemical stabilizing agents (surfactants such as CTAB, SDS etc., and polymers such as PVP) for preventing the self-aggregation of nanostructures [10-12]. The use of chemical compounds is toxic and will reduce the biological applicability. The use of natural products such as biosurfactant, monosaccharides, plant extracts etc. as enhancers and stabilizing agent for silver nanostructures synthesis were extensively studied. The marine glycolipid biosurfactant stabilized silver nanoparticles were synthesized by *Brevibacterium casei* MSA19 under solid state fermentation using agro-industrial and industrial waste as substrate [13]. Apte et al. [14] studied L-DOPA mediated synthesis of melanin by fungi *Yarrowia lipolytica* and the induced melanin has been exploited in the synthesis of silver and gold nanostructures. In this study, rapid reliable approach has been developed to produce uniform silver nanostructures by purified melanin from marine *Nocardiopsis alba* MSA10.

As melanin pigments are used as food colorant and nutritional supplements, which reflects the industrial need to large scale production as natural ingredients. Natural pigment production especially from microorganisms is emerging as an important aspect due to their wide acceptance in various industrial sectors [15] and it replaces the chemically synthesized pigments which cause harmful effects in the natural environment [16]. The microbial pigment, melanin has received considerable attention because of their useful biological activities especially in food and pharmaceutical industries. Melanins are high molecular weight pigments that are produced in microorganisms by oxidative polymerization of phenolic or indolic compounds with free radical generating and scavenging activity [17]. Based on chemical structure, properties and species affiliation, melanins are classified as allo-, pheo-, and eumelanins. The black or brown eumelanins are produced by oxidation of tyrosine through tyrosinase to DOPA (o- dihydroxyphenylalanine) and dopachrome, further the cyclization mediates to form 5,6-dihydroxyindole (DHI) or 5,6-dihydroxyindole-2-carboxylic acid (DHICA) [18]. The yellow-red pheomelanins are synthesized like eumelanins in the first step; the intermediate DOPA undergoes cysteinylation, directly or mediated by glutathione to form various derivatives of benzothiazines [19]. The third types of allomelanins are heterogenous group of polymers synthesized via pentaketide pathway [20]. Brown pigments may also produce from L-tyrosine pathway via accumulation and autooxidation of intermediates of tyrosine catabolism [18]). Microbial melanin has a wide range of applications including photoprotective, radioprotective, immuno-modulating, antimicrobial and antitumour activities [21-23]. Actinobacteria were resilient bacteria found among culturable sponge microbes and are current focus on bioactive leads from marine environment [24]. The sponge associated actinomycetes has wide application as antiviral, antibacterial, antitumour, anti-helminthic, insecticidal, immuno-modulator, immuno-suppressant and food colorants [25]. Melanin producing microorganisms are ubiquitous in nature; however limited literature is available on actinobacterial melanin production at different cultural conditions. Therefore, this study aims to enhance the production of melanin from marine actinobacterium *N. alba* MSA10, by optimizing various cultural and environmental parameters under submerged conditions as well as melanin mediated synthesis of silver nanostructures.

## Results

### Screening and identification of melanin producers

**Figure 1:**
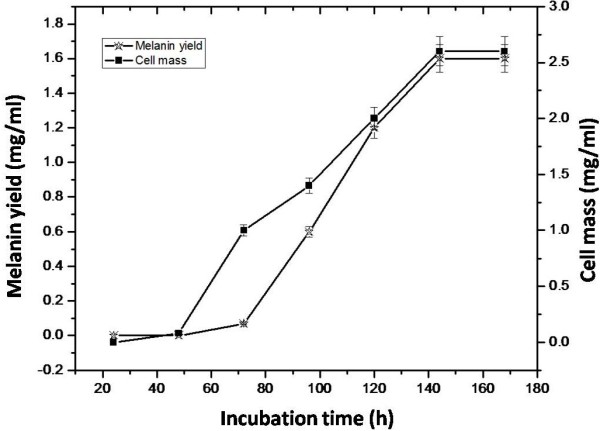
**Melanin production rate at different incubation time by *Nocardiopsis alba *** **MSA10.**

The strain MSA10 was considered as potential melanin producers among the other isolates obtained from the sponge *Dendrilla nigra.* The MSA10 strain was Gram positive and mycelia appearance under phase contrast microscope, which produce white powdery colonies on the actinomycetes isolation agar. It showed positive results on indole, citrate utilization, urease and triple sugar ion tests and negative results in methyl red, Voges Proskauer and catalase tests. Based on the morphological, biochemical, phylogenetic analysis (UPGMA algorithm) and taxonomic affiliation (RDP-II), the isolate MSA10 was identified as *Nocardiopsis alba* MSA10. The 16S rRNA sequence was deposited in Genbank with an accession number EU563352. It was found that the isolate MSA10 showed clustering exclusively with pigment producing *Streptomyces* strain and also an efficient biosurfactant producer [26]. Melanin production by *N. alba* MSA10 was initiated at 72 h of incubation, the medium changed to light brown, further the color development was increased at 96, 120 and 144 h to light brown, brown and dark brown respectively. The melanin production was depending on the biomass yield and a highest yield of biomass with melanin was obtained at 144 h of incubation (Figure 1).

### Formulation of fermentation media for melanin production

It is evident that different media constituents such as carbon, nitrogen, metal ions, and organic solvents and environmental factors such as pH, temperature, and salinity are known to play a vital role in the melanin production. The fermentation conditions and media constituents including sucrose, tyrosine, temperature and salinity as most significant variables were optimized for enhanced melanin yield. The correlation between melanin yield and the four critical control factors (variables) were analyzed by Box- Behnken design, the following quadratic model polynomial equation was obtained to explain melanin yield in mg/ml (Y).

(1) $$\begin{array}{ll} \;Y= & +3.40-0.092*A-0.042*B-0.13*C-0.15\\ & *D+0.12*A*B+0.000*A*C+0.20\\ & *A*D-0.100*B*C-0.10*B*D-0.30\\ & *C*D-0.67*A2-0.74*B2-0.75\\ & *C2-0.53*D2 \end{array}$$

The statistical significance of the equation 1 was checked by F- test and the results of ANOVA are shown in Table 1. The model F value of 251.68 implies the model is more significant (<0.0001). The coefficient determination (R^2^) value was found to be 0.9960, which implies that the variation of 99.60% for the melanin yield was attributed to the independent variables and only 0.40% of the total variation could not be explained by the model. The R^2^ value found in this study was closer to 1 show that the developed model could effectively increase the melanin production (3.4 mg/ml).

**Table 1:** ANOVA for response surface quadratic model of melanin production

**Source**	**Sum of squares**	**df**	**Mean square**	**F value**	**p-value prob > F**
**Model**	**9.02**	**14**	**0.64**	**251.68**	< 0.0001^**^
*A-Sucrose*	*0.10*	*1*	*0.10*	*39.40*	< 0.0001^**^
*B-Tyrosine*	*0.021*	*1*	*0.021*	*8.14*	0.0128^*^
*C-Temperature*	*0.21*	*1*	*0.21*	*83.35*	< 0.0001^**^
*D-Salinity*	*0.27*	*1*	*0.27*	*105.49*	< 0.0001^**^
*AB*	*0.063*	*1*	*0.063*	*24.42*	0.0002^*^
*AC*	*0.000*	*1*	*0.000*	*0.000*	*1.0000*
*AD*	*0.16*	*1*	*0.16*	*62.51*	< 0.0001^**^
*BC*	*0.040*	*1*	*0.040*	*15.63*	0.0014^*^
*BD*	*0.040*	*1*	*0.040*	*15.63*	0.0014^*^
*CD*	*0.36*	*1*	*0.36*	*140.65*	< 0.0001^**^
*A*^*2*^	*2.88*	*1*	*2.88*	*1126.34*	< 0.0001^**^
*B*^*2*^	*3.57*	*1*	*3.57*	*1394.02*	< 0.0001^**^
*C*^*2*^	*3.69*	*1*	*3.69*	*1441.40*	< 0.0001^**^
*D*^*2*^	*1.82*	*1*	*1.82*	*709.64*	< 0.0001^**^

^**^More significant, ^*^Significant; R-Squared 0.9960, Adj R-Squared 0.9921.

**Figure 2:**
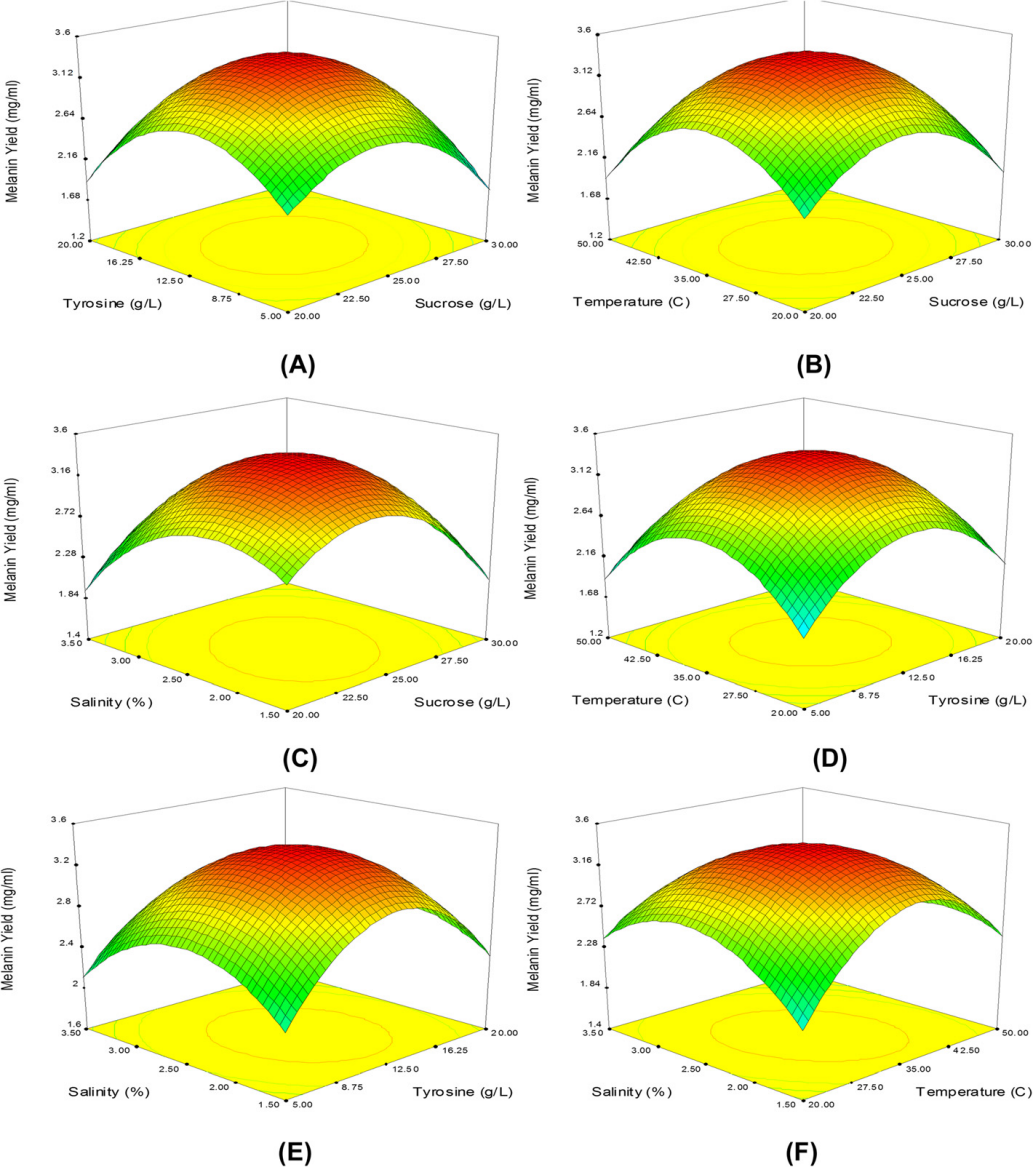
**The response surface plot shows the most significant variables interaction on melanin production. (A)** tyrosine and sucrose, **(B)** temperature and sucrose, **(C)** salinity and sucrose, **(D)** temperature and tyrosine, **(E)** salinity and tyrosine, and **(F)** salinity and temperature.

The 3D response surface plots showed the effect of medium components and fermentation conditions on the production of melanin (Figure 2). The response surface curve was plotted with two factors varied at a time when the other two factors as being remained at a fixed level. Higher melanin yield (3.4 mg/ml) was obtained with 12.5 g/l of tyrosine and 25 g/l of sucrose in the medium and maintaining the other parameters such as salinity (2.5%), pH (7.5) and temperature (35°C) as constant (Figure 2). When the pH was below 7.5 (6.0 – 6.5) and the temperature above 35°C, the growth of *N. alba* MSA10 as well as melanin production has declined drastically. The pigment production consequently increased with increasing the temperature up to 35°C, but the growth of *N. alba* MSA10 was found optimum at the temperature of 28 – 30°C. It was found that at pH 7.5, the growth of *N. alba* MSA10 and melanin production was found to be linear. This suggests that the near neutral pH was optimum for higher biomass and melanin production.

### Lights on melanin production

**Figure 3:**
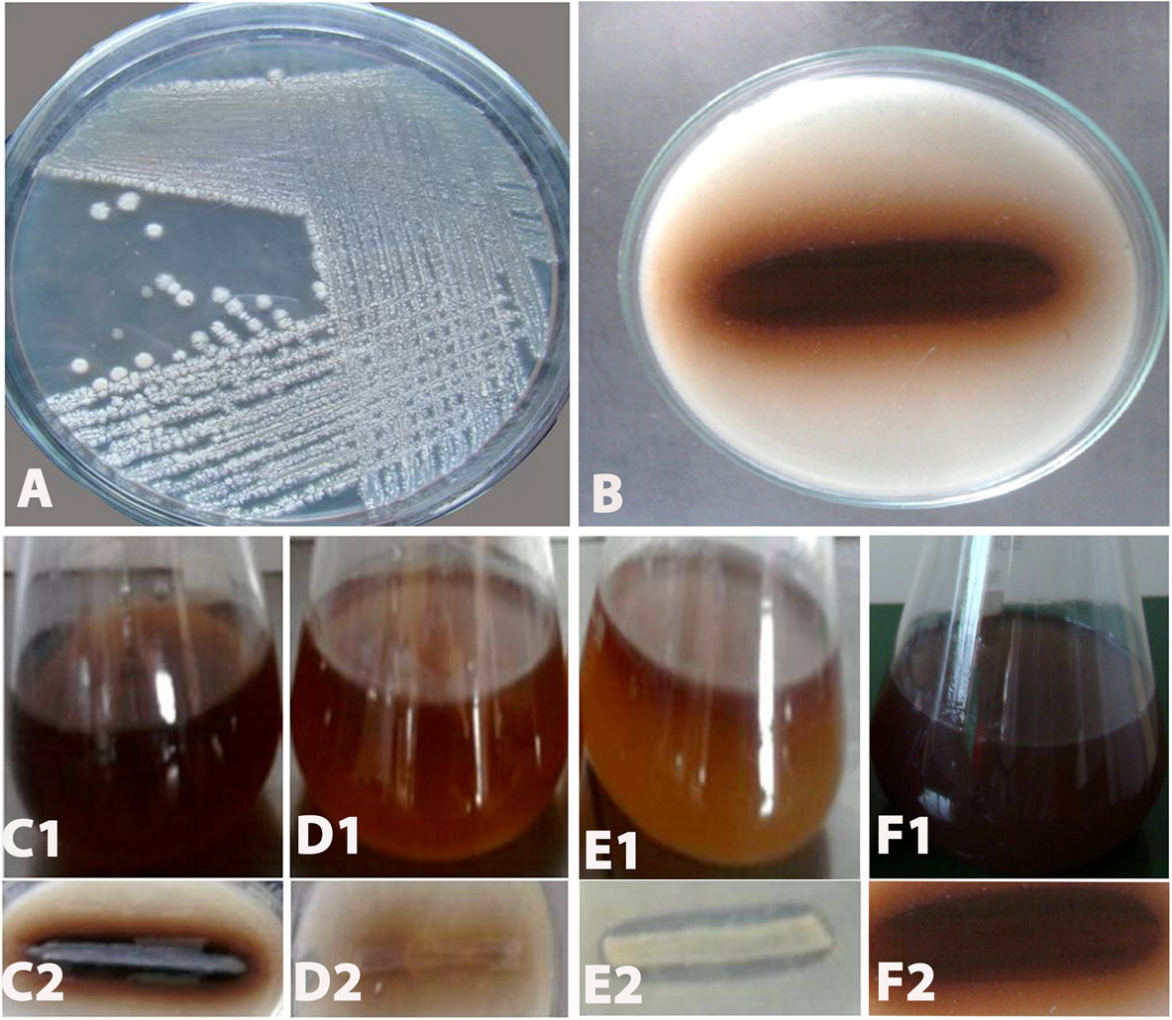
**Effects of light source on melanin production.** Growth of *N. alba* MSA 10 on Actinomycetes isolation agar medium **(A)**, Melanin production on tyrosine (1%) agar medium **(B)**, Green light source on melanin production in tyrosine broth **(C1)** and tyrosine agar medium **(C2)**, which produces dark brown pigment. Red light source produces brown pigment, tyrosine broth **(D1)** and tyrosine agar **(D2)**. Yellow light shows light brown pigment production on tyrosine broth **(E1)** at 144 h incubation, but no pigment production in tyrosine agar medium **(E2)**. The pigment production at normal light source on tyrosine broth **(F1)** and tyrosine agar medium **(F2)** produces dark brown pigment.

Light is considered as important environmental parameters for melanin production. Literature evidenced that pigments absorbed light at a particular wavelength and emits different colors. In this study, the various light sources such as green, red and yellow light on enhanced melanin production were investigated. It was found that the green light excitation had resulted in highest melanin production with the formation of dark brown color. Considerable pigment production was observed in red light and there is no pigment production in yellow light source in the culture plate, but slight production was observed in the fermentation medium at 144 h of incubation (Figure 3).

### Characterization of melanin pigment

The chromatogram of violet color spot on TLC plate showed an R_f_ value of 0.74 related to melanin pigment. A strong peak at 220 nm was obtained for UV- visible spectrum of *Nocardiopsis* melanin (data not shown). The colorimeter L* (lightness ranges from 0–100 (dark – light) a* (red- green) and b* (yellow-blue) values of melanin reflects the dark brown color. The L*, a* and b* values of melanin was found to be 2.74, 0.22 and 0.96 respectively. The low lightness value shows the dark color pigment. The values of a* (R-G) and b* (Y-B) represents the *Nocardiopsis* melanin pigment as a dark brown color. The FT-IR spectrum of column purified melanin showed the absorbance bands at 1118, 1385, 2077 cm^-1^ and a strong band at 3397, 1638, 674 cm^-1^. The intense broad band at 3397 cm^-1^ corresponds to the OH groups of polymeric structure, the band at 1638 and 1118 associated with primary amine NH and primary amine CN stretch vibrations of melanin respectively. The band at 1385 cm^-1^ is assigned to methylene scissoring of C-H groups and the band around 2077 arises from the carbonyl stretching vibrations. The TLC chromatogram and FT-IR spectrum analysis confirmed the melanin pigment produced by *N. alba* MSA10.

### Physico chemical properties of purified melanin

**Figure 4:**
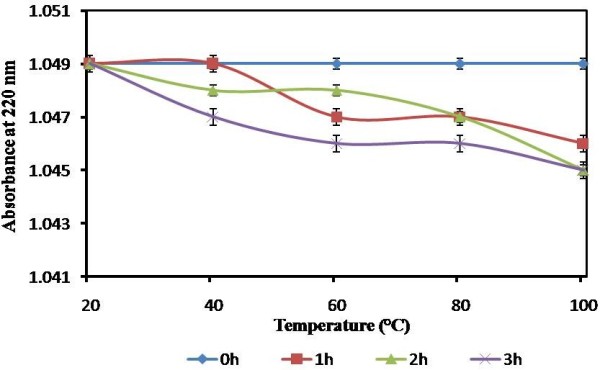
**Temperature stability of melanin produced by *Nocardiopsis alba *** **MSA 10 with different incubation times measured by UV absorbance at 220 nm (mean ± S.D., n = 3).**

The *Nocardiopsis* melanin was found to be dissolving immediately in alkaline water and hexane when compared to water at room temperature. A precipitation was formed when the melanin was allowed to dissolve in ethanol, methanol and HCl. It remains insoluble in ether, chloroform and ethyl acetate. *Nocardiopsis* melanin was stable at the range of temperatures (20–100°C) even for 3 h (Figure 4) and light sources including UV, natural sun light and complete darkness. The stability of different pH (3–12) of melanin tested had showed slight variation of absorption spectrum scanned at 190–220 nm (data not shown). The strong peak at 215 was observed (peak value 3.9) in the alkaline pH (9, 10 and 12), which indicates the relative stability of melanin in alkaline conditions when compared to neutral and acidic conditions. Similar water solubility nature of melanin has been reported in a mutant strain of *Bacillus thuringiensis*[27]. The other physico chemical properties are similar to melanin obtained from *Osmanthus fragrans* seeds [28].

### Antioxidant activity and reducing power of melanin

**Figure 5:**
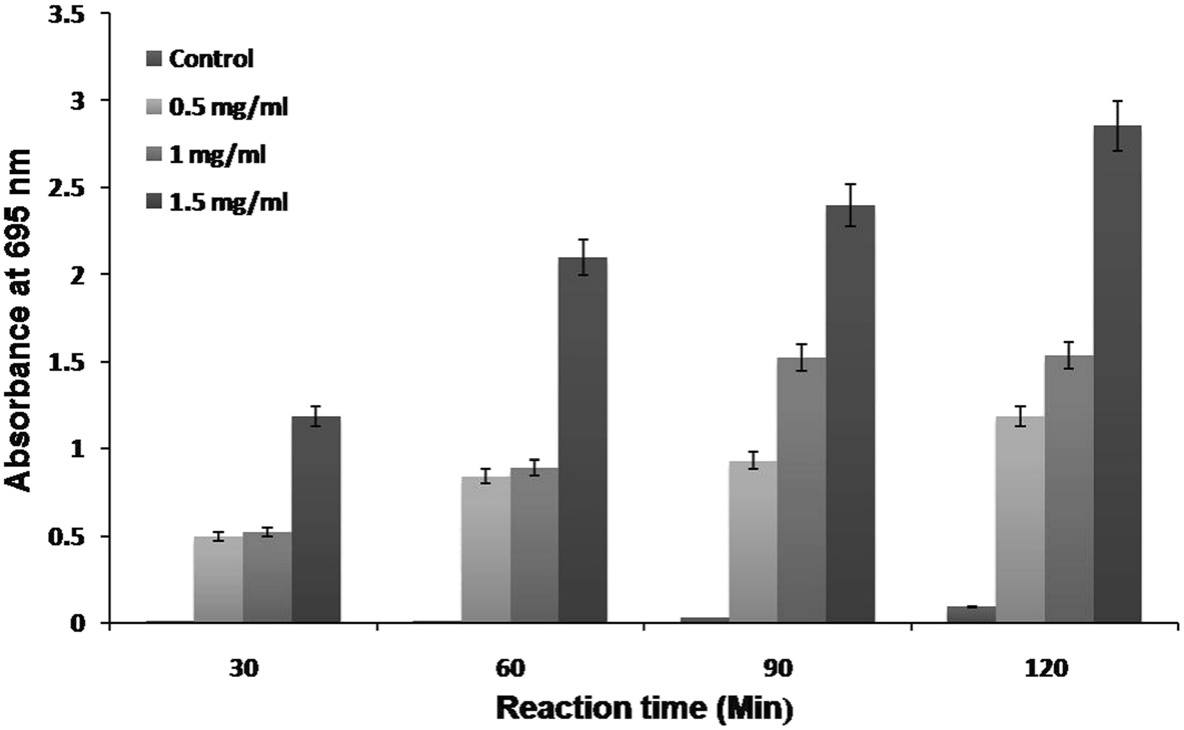
**Antioxidant activity in different concentration of *Nocardiopsis *** **melanin.**

**Figure 6:**
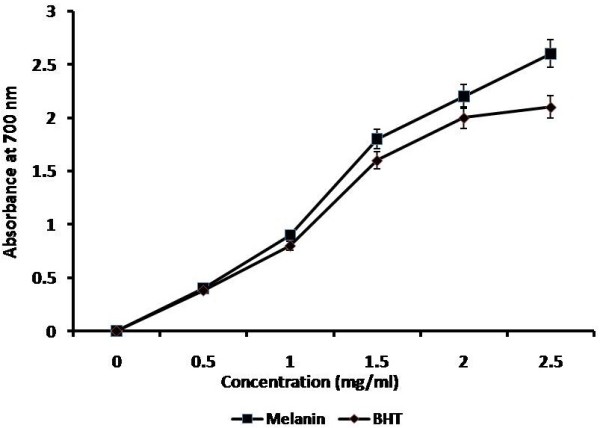
**Reductive capabilities in various concentrations of *Nocardiopsis *** **melanin and a standard BHT.**

The antioxidant assay is based on the reduction of Mo (VI) to Mo (V) by melanin with the formation of a green phospho molybdenum complex at different temperatures. Even though the green – complex formation takes place at room temperature, the formation of maximum phospho molybdenum complex increases with the increasing temperature of 90 and 180°C. Figure 5 shows the antioxidant property exhibited by *Nocardiopsis* melanin. Similar results were obtained with the melanin from berry of *Cinnamomum burmannii* and *Osmanthus fragrans*[29]. The reducing capabilities of *Nocardiopsis* melanin from Fe^3+^ to Fe^2+^ was clearly investigated with the standard BHT (Figure 6) and the results evidenced and validated the antioxidant property. Presence of antioxidant substances enhance the reduction of Fe^3+^/Ferricyanide complex to the Fe^2+^ form, which can be monitored at 700 nm [30].

### Melanin mediated synthesis of silver nanostructures and antimicrobial assay

**Figure 7:**
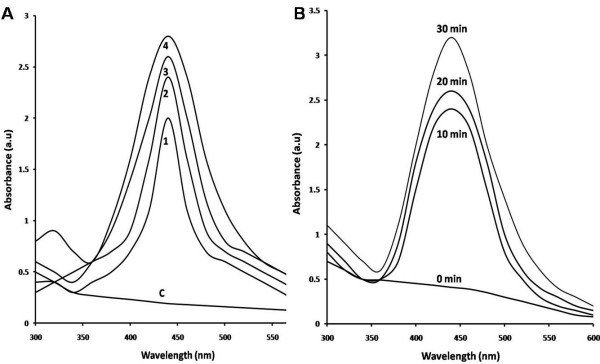
**UV absorption spectra of synthesized silver nanostructures. (A)** At different temperatures. C - Control (AgNO_3_ solution (1 mM), 1-40°C, 2-60°C, 3-80°C and 4-100°C **(B)** At different time intervals.

**Figure 8:**
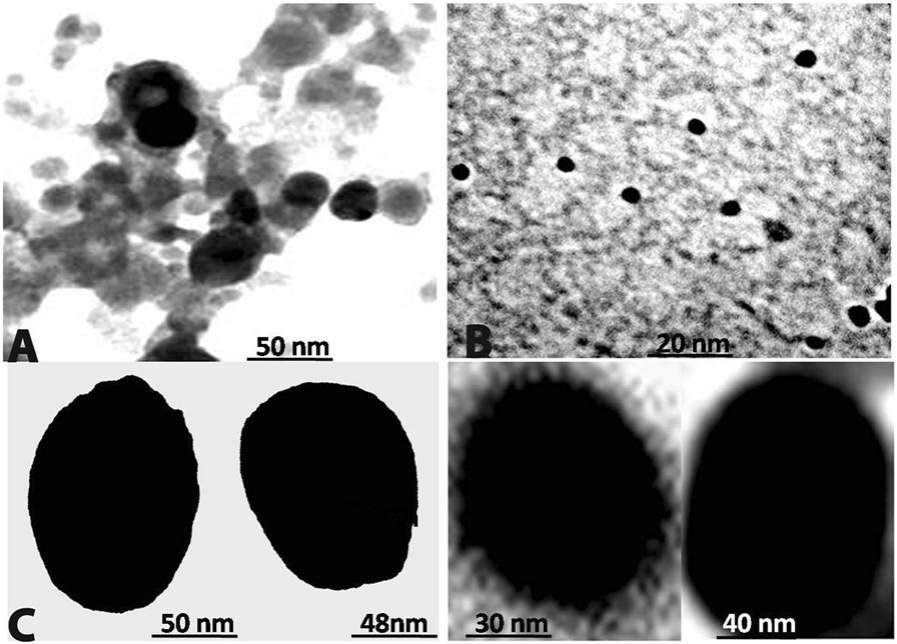
**TEM images of synthesized silver nanostructures. (A)** Silver nanostructures, **(B)** nanostructure synthesis at 100°C and **(C)** different sizes of synthesized silver nanostructures.

**Figure 9:**
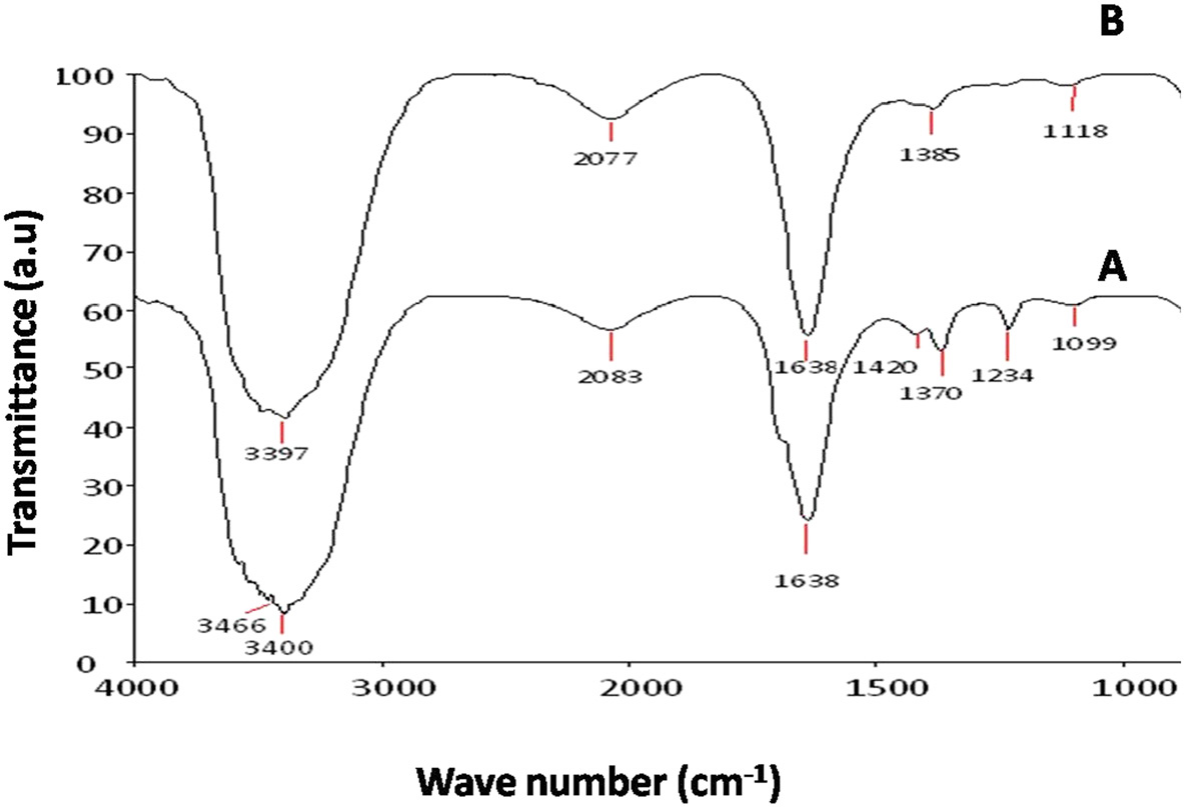
**FTIR spectra of synthesized silver nanostructures. (A)** Silver nanostructures, and **(B)** pure melanin.

**Figure 10:**
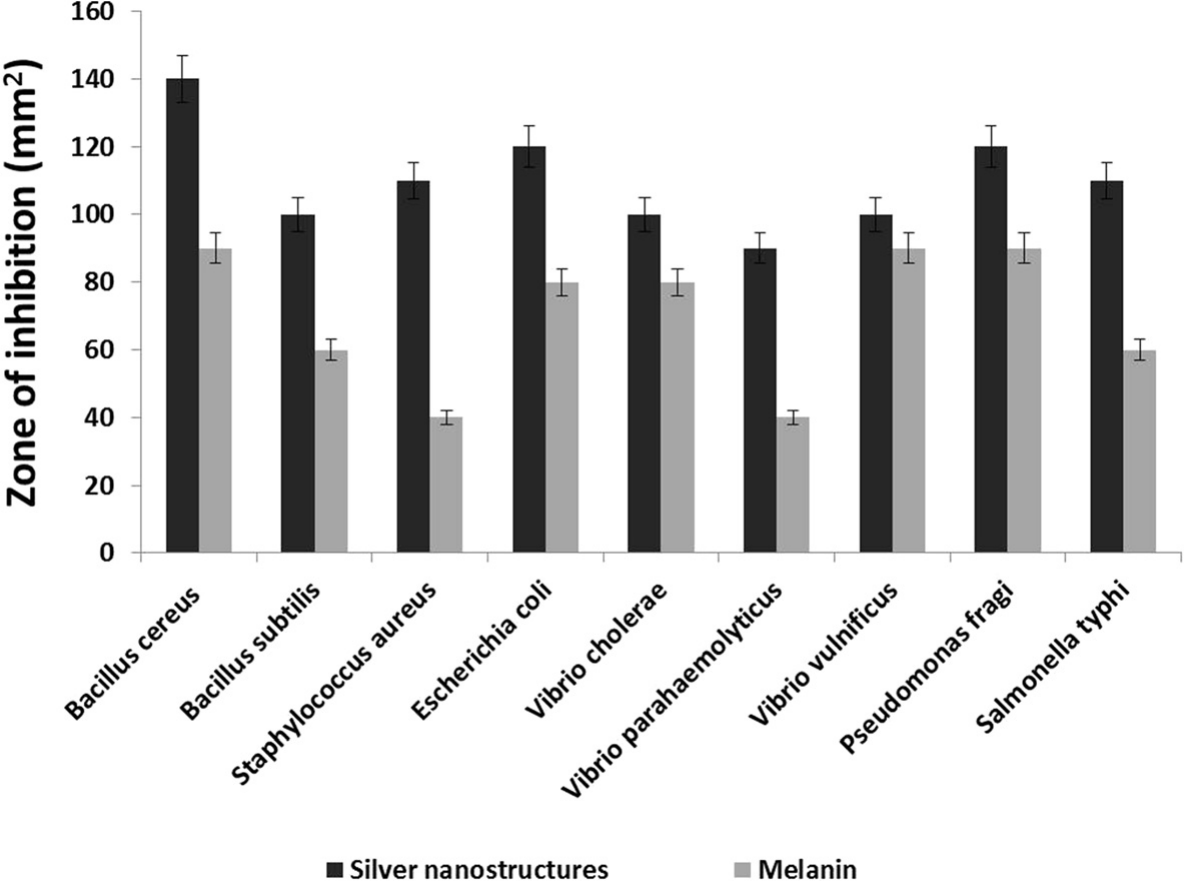
Antimicrobial assay of melanin and melanin- mediated synthesis of silver nanostructures.

The synthesis of melanin mediated silver nanostructures was confirmed by the appearance of strong peaks in the UV- visible spectra at 420–460 nm. With the increase in temperature (100°C) stable and rapid synthesis of same sized particles takes place (Figures 7A and 8B). The synthesis pattern of UV- Visible spectrum at different temperature profile is depicted in Figure 7B. It is evident from the UV – absorbance spectrum that the temperature at 100°C shows effective synthesis. It is noticeable that the temperature stability of *Nocardiopsis* melanin tested before showed stability at 100°C over 3 h. The antioxidant and reductive capabilities of the melanin compound enhances the rapid synthesis of silver nanostructures without adding any capping agent. Thus, melanin acts as both reducing and capping agent of silver nano- sized structures synthesis. The synthesis at various time interval shows that increasing incubation time at 30 min gives more stable particles when compared to 0, 10 and 20 min (Figure 7B). The FT-IR spectrum of melanin mediated silver nanostructures shows (Figure 9) characteristics absorbance bands of 3466, 3400, 2083, 1638, 1420, 1370, 1234, 1099 and 664 cm^-1^ respectively. The shift in the bands at 1118 of pure *Nocardiopsis* melanin towards their lower frequency to 1099 is attributed to the binding of primary amine (N-H) to the silver ions. The shifting of symmetrical stretch of carboxylate group at 1385 to 1370 and 1234 clearly shows the reaction between silver particles to carboxylate group of melanin. The appearance of new band at 3466, shifting of band at 3397–3400 and 2077–2083 cm^-1^ were suspected to cause heating of melanin with silver nitrate solution. The TEM results (Figure 8) showed that all synthesized particles were spherical in shape and found to be well dispersed in aqueous medium. The particle sizes ranging from 20 – 50 nm were formed. The melanin-silver nanoparticles showed (Figure 10) antimicrobial activity against all food pathogens tested but the highest activity was found against *B. cereus* (140 mm^2^) and *P. fragi* and *E. coli* (120 mm^2^ respectively).

## Discussion

Industrial production of colorants from microorganisms are more suitable due to factors such as ease of availability, culturing, higher production of pigments and the microbes’ potential to be genetically manipulated. Isolation of new strain is still of particular interest because of the necessity to obtain microorganisms with suitable characteristics for submerged cultivation. Recently, sponge associated marine bacteria have been considered as a potential source of food- grade pigments [31].

The production of melanin by *N. alba* MSA10 was attributed to the supplement of tyrosine on the production medium via tyrosinase enzyme. The formation of dopachrome (red coloration) and the OD of 0.148 in tyrosinase assay were confirmed by tyrosinase activity of *N. alba* MSA10. The strain *N. alba* MSA10 utilized up to 12.5 g/l of tyrosine but on further addition up to 20 g/l, the melanin production rate gets declined. This shows that the strain *N. alba* MSA10 had produced melanin by the mediation of tyrosinase. According to Williams [32], about one third of the taxa of the genus *Streptomyces* produce melanin. In strains including *Streptomyces antibioticus*, *S. glaucescens* and *S. lavendulae*, the tyrosinase gene for melanin production have been cloned, sequenced and recombinantly produced the protein which has sequence similarity to mammalian tyrosinase [33,34]. Melanin like pigments formed from L-tyrosine with different melanogenic pathway in *S. avermitilis*[35], *Xanthomonas campestris*[36], *Shewanella colwelliana*[37] and *Vibrio cholerae*[38] has been well deliberated.

Sucrose (25 g/l) as carbon source increased the melanin production up to 3.4 mg/ml significantly followed by glucose as alternative carbon source in *N. alba* MSA 10. Till date, there is no report on the production of melanin from sucrose as a sole carbon source. The red pigment produced by *Paecilomyces sinclarii* showed maximum mycelial growth in sucrose as carbon source, even though the highest pigment production has been attributed in soluble starch medium [39].

Stimulatory effects of various nitrogen sources including peptone, beef extract, yeast extract, urea and ammonium nitrate were tested by Placket-Burman experimental design, and the significant effect was found in case of beef extract and ammonium nitrate on melanin production. This shows that only trace amount of nitrogen source was utilized by *N. alba* MSA10 for melanin production, as the amino acid tyrosine mediates the melanin synthesis pathway. The strain MSA10 had utilized considerable level of nitrogen sources for their growth and mycelial development; however melanin production gets enhanced with the addition of tyrosine in the production medium. The strain grows optimum up to 3.5% of NaCl and the highest melanin production (3.4 mg/ml) has obtained at 2.5% of salinity. Further increasing salinity, the melanin production was found to be decreased. Melanin production by *N. alba* MSA10 was highest at 35°C and pH 7.5. The highest yield of pigment from *Monascus* was reported at 30°C [40]. The initial pH at 6 and temperature of 32°C increased the pigment production by *Monascus* sp. [41].

The pigment production by *Monascus purpureus* with various light sources was well recognized by Babitha et al.[42] and this finding described that red light have little effect on growth and pigment production when compared to green and blue light sources which probably inhibits the pigment production, even though there is significant increase in biomass under green light. Despite the importance of influence of light on pigment production as investigated on *Monascus purpureus*[42], much has not yet been determined on actinomycetes melanin. Therefore, the strain *N. alba* MSA10 would be the first record among the actinomycetes produced melanin under illumination of the green light source.

The predicted melanin yield was found to be closer to actual melanin yield and the production rate was increased one fold over the wild strain *N. alba* MSA10. It reveals that the generated Box- Behnken design showed the interaction and actual relationships between the critical control factors. The RSM-based experiments showed that *N. alba* MSA10 has higher melanin (3.4 mg/ml) productivity potential.

The FT-IR absorbance band of *Nocardiopsis* melanin ranging from 3400 cm^-1^ to 674 cm^-1^ had high degree of similarity to the BC58 melanin, standard melanin sigma [43] and synthetic pyomelanin, pyomelanin extracted from *Aspergillus fumigatus*[44]. The shifting of band related to primary amine (N-H), carboxylate group and C-O stretch vibration clearly evidenced that *Nocardiopsis* melanin reduces silver nitrate and at the same time it stabilizes the synthesized silver nanostructures. The stability of melanin mediated silver nanostructures were determined by synthesized particles which was allowed to stands for 3 months at room temperature. It was found that the color intensity of silver particles increased with aging and no aggregation was observed in duration of 3 months. The free amine or carboxylate group of proteins can bind with silver particles [45]. The interaction of melanin with metal ions, protein [46] and double stranded DNA [47] was extensively studied.

The melanin mediated silver nanostructures found to be most effective on food pathogens such as *B. cereus*, *P. fragi* and *E. coli*. Thereby, the incorporation of melanin mediated synthesized silver nanostructures in food packaging materials can effectively inhibit the growth of food pathogens and increase the shelf life of packed food products. Nanomaterials are being explored for their promising role in food industry such as providing longer shelf-life for foods, better barrier properties, improved heat resistance and temperature control, and anti-microbial and fungal protections [48]. Silver nanoparticles that act as antibacterial agents or nanoclay coatings are currently used in food packaging [49].

The future studies can be focused on the rapid formation of different shape of silver nanostructures under optimal conditions with melanin as reducing and capping agent. The size and shape based silver nanostructures has many positive attributes such as good conductivity, chemical stability, catalytic and antimicrobial activity that make them suitable for many practical food packaging applications. The melanin mediated silver nanostructures can be incorporated in food packaging materials. The efficacy of melanin-silver nano-conjugates on the shelf life of packed food products is needs to be investigated.

## Conclusion

The melanin pigment has been successfully purified and characterized from *N. alba* MSA10. The cultural conditions and environmental factors for enhanced yield of melanin were optimized through RSM- Box- Behnken design. The purified melanin has been used to synthesize and stabilizes the silver nanostructures *in vitro*. The antioxidant activity, reducing power and physico-chemical properties of *Nocardiopsis* melanin was well characterized. The antioxidant, antimicrobial and natural coloring potential of *Nocardiopsis* melanin can be used as food additives, which significantly reduces the usage of artificial or synthetic colorants and antioxidants. The UV protective roles, withstanding higher temperatures, stability in alkaline conditions and water solubility nature of *Nocardiopsis* melanin increased their application in food, cosmetics and biomedical industries. Thus, the synthesis and stabilization of silver nanostructures by *Nocardiopsis* melanin demonstrates the metal interacting nature of pigment. Furthermore the antibacterial properties against food pathogens would facilitate its applicability in food processing and food packaging industries.

## Methods

### Isolation, screening and identification of melanin producing marine actinobacterium

The marine actinobacteria were isolated from marine sponge *Dendrilla nigra* as described by Selvin et al. [50]. The isolated actinobacteria were screened for melanin production on tyrosine agar medium (g/l): (peptone 5 g, sodium chloride 20 g, Beef extract 1.5 g, yeast extract 1.5 g, tyrosine 10 g, agar 20 g and pH 7.3) and were incubated at 30–35°C for 6–7 days. The pigment production was confirmed by the formation of a brownish color around the colonies. The melanin producer strains were identified based on morphological, biochemical and phylogenetic analysis [50].

### Fermentation by shake flask culture

The melanin production was carried out in five sets of 250 ml Erlenmeyer flasks under shake flask culture containing 100 ml of tyrosine medium. The culture flasks were incubated at 35°C for 7 days on a rotary shaker (Oasis) at 200 rpm. Samples were removed after initial color change periodically for biomass and melanin yield determination. The biomass yield was estimated by washing the cells with phosphate buffered saline (g/l) (NaCl 8 g, KCl 0.20 g, Na_2_HPO_4_ 1.44 g, KH_2_PO_4_ 0.24 g, pH 7.4) and dried at 50–60°C for 2 h. The melanin supernatant was first adjusted to pH 9 with 10 N NaOH to ensure polymerization and then adjusted to pH 3 with 5 N HCl to precipitate melanin. The precipitated melanin was centrifuged at 10,000 rpm for 15 min (Eppendorf), washed thrice with deionized water and lyophilized for dry weight determination.

### Formulation of fermentation media for melanin production

To formulate the media with various concentrations of media constituents on melanin production by MSA10, different carbon, nitrogen sources, metal ions and organic solvents were used. The carbon sources used in this study include 20 g/L of glucose, dextrose, sucrose, mannitol and galactose. The organic nitrogen sources include 15 g/L of peptone, yeast extract, beef extract, and inorganic nitrogen sources of urea and ammonium nitrate are at the concentration of 100 mg/L. The pH of the melanin pigment production was studied using shake flask cultures at different initial values of pH (4–10). The effect of temperature on pigment production was determined with different incubation temperatures (25–60°C). The NaCl requirement for pigment production was optimized with 0.5 to 3.5% NaCl supplementation. Different metal ions such as CuSO_4_, FeSO_4_, MgSO_4_, MnCl_2_ and MnSO_4_ were added in tyrosine broth at 100 mg/L concentration to determine the effect of metal ions.

### Experimental design and statistical analysis

For all experiments, fermentation was conducted in 500 ml of Erlenmeyer flasks containing different media constituents on melanin production. All experiments were carried out in triplicate and the final melanin yield was taken as the response (y). The Box-Behnken experimental design with four variables (A, B, C and D) such as sucrose, tyrosine, temperature and salinity respectively and three levels high (+), middle (0), and a low (-) was employed to optimize the fermentation conditions and thereby to obtain maximum melanin yield. The experimental design with four variables is summarized in Table 2. Based on Placket-Burman experimental design, the most significant variables sucrose, tyrosine, temperature and salinity were identified from the 11 variables analyzed such as glucose, sucrose, yeast extract, mannitol, tyrosine, ammonium nitrate, ferrous sulphate, pH, temperature, salinity and inoculums size (data not shown). The experimental data was analyzed using the software Design expert 8.0.4.1 trial version (Stat-Ease, Inc, USA).

**Table 2:** Box - Behnken experimental design with four independent variables (coded values) and the melanin production rate

**Run**	**A**	**B**	**C**	**D**	**Melanin yield (mg/ml)**	
					**Actual value**	**Predicted value**
1	0	-1	1	0	1.90	1.94
2	1	0	0	-1	2.00	1.98
3	-1	0	0	1	2.00	2.06
4	0	0	1	-1	2.40	2.34
5	0	1	-1	0	2.10	2.12
6	1	-1	0	0	1.80	1.82
7	-1	-1	0	0	2.20	2.25
8	0	-1	-1	0	2.00	1.98
9	-1	1	0	0	1.90	1.91
10	0	1	0	-1	2.40	2.43
11	0	1	0	1	1.80	2.40
12	-1	0	-1	0	2.20	2.16
13	1	1	0	0	2.00	1.96
14	0	0	0	0	3.40	3.40
15	0	0	1	1	1.50	1.53
16	-1	0	1	0	2.00	1.95
17	0	0	-1	1	2.40	2.43
18	0	-1	0	1	2.10	2.14
19	0	1	1	0	1.60	1.63
20	-1	0	0	-1	2.60	2.65
21	0	0	0	0	3.40	3.40
22	0	0	0	0	3.40	3.40
23	0	0	0	0	3.40	3.40
24	1	0	0	1	2.20	2.16
25	0	-1	0	-1	2.30	2.22
26	0	0	0	0	3.40	3.40
27	0	0	-1	-1	2.10	2.10
28	1	0	-1	0	2.00	2.02
29	1	0	1	0	1.80	1.75

A: Sucrose -1(20 g/l), 0 (25 g/l), +1(30 g/l); B: Tyrosine, -1(5 g/l), 0 (12.5 g/l), +1(20 g/l); C: Temperature, -1(20°C), 0(35°C), +1(50°C); D: Salinity, -1(1.5%), 0(2.5%), +1(3.5%).

### Light source on melanin production

The effect of light on melanin production by MSA 10 was studied by passing different wavelengths of light, red (620 – 750 nm), blue (450–475 nm), green (495–570 nm) on fermentation medium. The culture flasks were exposed to the light intensity of 32 W m^-2^ for 7 days.

### Assay for tyrosinase activity

Tyrosinase activity was assessed by growing the MSA10 isolates in to glutamate medium [51] and 2 ml of culture supernatant mixed with 2 ml of 0.1 M phosphate buffer (pH 5.9), finally 1 ml DOPA was (10 mM) added. The reaction mixture was incubated at 37°C for 5 min. Red coloration resulting from dopachrome formation was observed and read spectrophotometrically at 475 nm (PG Instruments).

### Characterization of melanin pigment

The cell free supernatant was collected from fermented broth by centrifugation at 10,000 g for 15 min (Eppendorf 5804 R). The supernatant was filtered through Whatman No.1 filter paper to remove residue cell debris. The initial purification of melanin was performed according to Wan et al. [20]. Briefly, the melanin supernatant was first adjusted to pH 9 with 10 N NaOH to ensure polymerization and then adjusted to pH 3 with 5 N HCl to precipitate melanin. The precipitated melanin was centrifuged, washed thrice with deionized water and lyophilized for further use. The absorbance spectrum of melanin produced by MSA10 was measured with UV/VIS Spectrophotometer (PG instruments) over a range of wavelengths from 190 to 500 nm. The color intensity of melanin was measured by CR- 300 colorimeter with the HunterLab color system. The L* (lightness ranges from 0–100 (dark – light)), a* (red- green) and b* (yellow-blue) values were determined. The lyophilized melanin pigment was spotted on the TLC plate and the chromatogram was performed with the solvent system *n*-butanol: acetic acid: water (70:20:10). After drying, the pigment spot was sprayed with ninhydrin. The TLC purified pigment were applied to a column of DEAE-Cellulose (Bio-Rad, 1 × 30 cm) that had been equilibrated with 25 mM Tris–HCl buffer (pH 8.6) containing 50 mM sodium chloride. The column was eluted at a flow rate of 100 ml/h with 1:1 volume gradient from 0.1 M to 2 M NaCl in the same buffer.

### Physico-chemical properties of the melanin

The physico – chemical properties of *Nocardiopsis* melanin was analyzed according to Wang et al. [21]. The solubility of purified melanin was checked by adding 0.05 g of the melanin in 10 ml of water, aqueous acid, alkali (such as Na_2_CO_3_, NaOH solution), and organic solvents such as chloroform, ethyl acetate, ethanol, methanol, acetic acid, petroleum ether, hexane with stirring at 25°C for 1 h, then filtered and the absorption of the solutions were recorded spectrophotometrically at 220 nm. The temperature stability of melanin pigment was measured after treatment with various temperatures in a thermostatically controlled water bath at 20, 40, 60, 80 and 100°C for 3 h and subsequently the absorption of the solutions were recorded at 220 nm. Light stability of melanin was detected by holding the melanin solution (5 mg/ml) under natural light, at dark place and under the Ultraviolet-light far from 30 cm for two days and every 12 h interval the maximum absorbance was measured at 220 nm. The pH stability was assessed by adjusting the melanin solution (5 mg/ml) in to a varied pH range (3, 4, 6, 7,9,10 and 12) with 0.5 N NaOH and HCl. All the samples were held for 30 min at 25°C, and the absorption spectrum (190–220 nm) was scanned.

### Determination of antioxidant activity and reducing power of melanin

The antioxidant activity of *Nocardiopsis* melanin was determined by a standard spectroscopic method [52]. Briefly, Aliquots of 2 ml of different concentration of melanin solution (0.5, 1 and 1.5 mg/ml) prepared in phosphate buffer (0.2 M, pH 6.6) and mixed with 2 ml of reagent solution (0.6 M sulfuric acid, 28 mM sodium phosphate, and 4 mM ammonium molybdate). The tubes were capped and incubated in a thermal block at 95°C for 120 min. Every 30 min the absorbance of the mixture was measured at 695 nm against a blank.

The reducing power of the melanin pigment was determined by standard method [53]. Briefly, different concentrations of melanin were mixed with phosphate buffer (2.5 ml, 0.2 M, pH 6.6) and potassium ferricyanide [K_3_Fe (CN)_6_] (2.5 ml, 1%). The mixture was incubated at 50°C for 20 min. 2.5 ml of TCA (10%) was added to the mixture, which was then centrifuged at 3000 rpm for 10 min. The supernatant (1.0 ml) was mixed with distilled water (7.0 ml) and FeCl_3_ (0.5 ml, 0.1%), and the absorbance was measured at 700 nm. The Butylated hydroxytoluene (BHT in ethanol solution) was used as the standard and the obtained value was used to compare and interpret the result with melanin.

### Synthesis of melanin mediated nanostructures by boiling method

Silver nanostructures were synthesized *in vitro* by adding 10 ml purified melanin solution (20 μg/ml) to 40 ml of 1 mM AgNO_3_ (Sigma) and vigorously stirred for 5 minutes. The mixture was incubated at 60°C for 30 min. Both melanin and AgNO_3_ was maintained separately as control. Silver nanostructures synthesis at different temperature range from 40–100°C and different time intervals (0- 30 min) were studied at 1 mM AgNO_3._ Then the nanostructures were characterized by UV–vis spectrophotometer (PG instruments), FT-IR spectrum (Spectrum RX1) and TEM analysis. TEM measurements were performed on a TECHNAI 10 PHILIPS model instrument operating at an accelerating voltage of 80 kV.

### Antimicrobial assay of melanin and silver nanostructures against food pathogens

The silver nanostructures and the column purified melanin compound were tested for antimicrobial activity using well diffusion method and the area of the halo was measured [54]. The synthesized nanostructures were tested against common food pathogens such as *Bacillus subtilis* (MTCC 1305), *Bacillus cereus* (MTCC 1307), *Staphylococcus aureus* (MTCC 2940), *Escherichia coli* (MTCC 739)*, Vibrio cholerae* (MTCC 3906), *Vibrio parahaemolyticus* (MTCC 451), *Vibrio vulnificus* (MTCC 1145)*, Pseudomonas fragi* (MTCC 2458) and *Salmonella typhi* (MTCC 734). These were cultured on Muller Hinton agar (Himedia). Well was made with a sterile steel cork borer (1 cm diameter) and 50 μl of purified melanin and silver nanostructures were added in the wells, incubated at 30°C for 24 h. After incubation the clear halo was measured and the area of inhibition in mm^2^ was calculated.

## Abbreviations

MSA: Marine sponge associated actinobacteria; UPGMA algorithm: Unweighted pair group method with arithmetic mean algorithm; RDP-II: Ribosomal database project; ANOVA: Analysis of variance; TLC: Thin layer chromatography; UV- visible spectrum: Ultra violet – visible spectrum; FT-IR: Fourier transform infrared; BHT: Butylated hydroxytoluene; TEM: Transmission electron microscopy; OD: Optical density; RSM: Response surface methodology.

## Competing interests

The authors declare that they have no competing interests.

## Authors’ contributions

DA and ANL performed the experiments and GSK wrote the manuscript. MVA and NAA set the rationale of the experiments. JS designed the study and helped for preparing the manuscript. All authors read and approved the final manuscript.
